# YBX1 Promotes Esophageal Squamous Cell Carcinoma Progression via m5C‐Dependent SMOX mRNA Stabilization

**DOI:** 10.1002/advs.202302379

**Published:** 2024-04-02

**Authors:** Liwen Liu, Yu Chen, Tao Zhang, Guangying Cui, Weiwei Wang, Guizhen Zhang, Jianhao Li, Yize Zhang, Yun Wang, Yawen Zou, Zhigang Ren, Wenhua Xue, Ranran Sun

**Affiliations:** ^1^ Precision Medicine Center The First Affiliated Hospital of Zhengzhou University Zhengzhou Henan 450052 China; ^2^ Academy of Medical Sciences Zhengzhou University Zhengzhou Henan 450052 China; ^3^ Department of Oncology The First Hospital of Lanzhou University Lanzhou Gansu 730000 China; ^4^ Department of Pathology The First Affiliated Hospital of Zhengzhou University Zhengzhou Henan 450052 China; ^5^ Department of Pharmacy The First Affiliated Hospital of Zhengzhou University Zhengzhou Henan 450052 China

**Keywords:** 5‐methylcytosine, ESCC, metastasis, SMOX, YBX1

## Abstract

The modification and recognition of 5‐methylcytosine (m5C) are involved in the initiation and progression of various tumor types. However, the precise role and potential mechanism of Y‐box‐binding protein 1 (YBX1) in esophageal squamous cell carcinoma (ESCC) remains unclear. Here, it is found that YBX1 is frequently upregulated in ESCC compared with matched nontumor tissues. Gain‐ and loss‐of‐function assays show that YBX1 promoted the proliferation and metastasis of ESCC cells both in vitro and in vivo. Functional studies revealed that NOP2/Sun RNA methyltransferase family member 2 (NSUN2) is a critical RNA methyltransferase that facilitates YBX1‐mediated ESCC progression. Mechanistically, integrated analysis based on RNA immunoprecipitation sequencing (RIP‐seq) and m5C methylated RNA immunoprecipitation and sequencing (MeRIP‐seq) assays identified spermine oxidase (SMOX) as a target gene containing an m5C site in its coding sequence (CDS) region, which coincided well with the binding site of YBX1. Overexpression of SMOX‐WT but not SMOX‐Mut partially restored the proliferation and invasion ability of ESCC cells curbed by YBX1 knockdown. Moreover, YBX1 activated the mTORC1 signaling pathway by stabilizing *SMOX* mRNA. The study reveals that YBX1 promotes ESCC development by stabilizing *SMOX* mRNA in an m5C‐dependent manner, thus providing a valuable therapeutic target for ESCC.

## Graphical abstract

Liu et al. identify that YBX1, an m5C reader protein, is upregulated in ESCC tissues. Further, YBX1 promotes ESCC progression by stabilizing SMOX mRNA and consequently activating the mTOCR1 signaling pathway in an NSUN2‐mediated m5C modification‐dependent manner. This finding provides a preclinical rationale for selectively targeting YBX1‐mediated m5C recognition as a promising therapeutic strategy for ESCC.

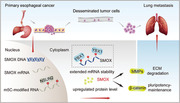

## 1 Introduction

Esophageal cancer is one of the most aggressive gastrointestinal malignancies globally.^[^ ^1^ ^]^ Among primary esophageal cancers, esophageal squamous cell carcinoma (ESCC) is the most prevalent subtype in China.^[^ ^2^ ^]^ Despite great advances in therapy, the overall 5‐year survival rate of patients with ESCC remains below 20% owing to its high invasiveness and recurrence rates.^[^ ^3^ ^]^ Hence, it is important to explore the mechanisms underlying the pathogenesis of ESCC to identify effective therapeutic targets.

Dynamic RNA modifications have emerged as critical post‐transcriptional regulators for tuning genetic information during embryonic development and disease progression.^[^ ^4^ ^]^ Recently, 5‐methylcytosine (m5C), an RNA modification that usually exists in tRNAs and rRNAs, has gained attention for its critical regulatory role in mRNA metabolism.^[^ ^5^ ^]^ Generally, the RNA m5C was thought to be mainly written by NOP2/Sun RNA methyltransferase family member 2 (NSUN2), a crucial m5C “writer” that participates in a variety of biological processes.^[^ ^6^ ^]^ Nevertheless, the final effect of posttranscriptional regulation mostly depends on the m5C “readers”, which recognize the m5C modification and critically affect the export, stability, and translation initiation of mRNAs.^[^ ^6^, ^7^ ^]^ For instance, Y‐box‐binding protein 1 (YBX1) is a well‐established m5C reader. It modulates the stability of target transcripts by recognizing the m5C modification.^[^ ^7a^ ^]^ Previous studies have demonstrated the oncogenic function of YBX1 as an RNA‐binding protein in tumorigenesis and cancer metastasis such as hepatocellular carcinoma (HCC),^[^ ^8^ ^]^ colorectal cancer,^[^ ^9^ ^]^ Chronic myeloid leukemia^[^ ^10^ ^]^ and clear cell renal cell carcinoma.^[^ ^11^ ^]^ Recently, increasing evidence has demonstrated that YBX1 functions as an m5C reader in bladder,^[^ ^7a^ ^]^ breast,^[^ ^12^ ^]^ and gynecologic cancers.^[^ ^13^ ^]^ However, the role of YBX1 in the progression of ESCC remains unclear.

In this study, we verified that YBX1 was markedly upregulated in ESCC. Functionally, YBX1 promoted the proliferation and metastasis of ESCC cells. Mechanistically, we found that YBX1 preferentially bound to spermine oxidase (SMOX) mRNA and enhanced its stability in a NSUN2‐mediated m5C‐dependent manner. Our current study illustrates a novel molecular mechanism of m5C‐mediated oncogene activation during ESCC progression and provides a rationale for targeting the YBX1/m5C‐SMOX signaling axis for the treatment of ESCC.

## 2 Results

### 2.1 YBX1 Expression was Elevated in ESCC Tissues and Associated with Poor Survival

**Figure 1:**
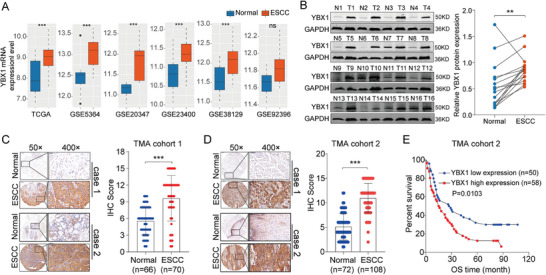
YBX1 was upregulated in ESCC tissues and associated with patients poor survival. A) The mRNA expression level of YBX1 in ESCC tissues was analyzed in TCGA and GEO datasets. B) The protein expression status of YBX1 in 16 pairs ESCC tissues and adjacent normal esophageal tissues was determined by western blot (left) and further quantified (right). C,D) IHC analysis of YBX1 in ESCC TMA cohorts. E) Kaplan–Meier survival analysis of YBX1 in ESCC patients (ESCC TMA cohort 2; n = 108). ns, none significance; ^**^ *p* < 0.01; ^***^ *p* < 0.001.

To explore the potential role of YBX1 in esophageal cancer, we first investigated the expression profile of YBX1 based on public datasets and further validated it using our own clinical ESCC samples by western blotting. We found that the expression of YBX1 was significantly upregulated in ESCC tissues (**Figure** **1**A,B). We also performed IHC staining in samples from two independent TMA cohorts and further confirmed the high expression of YBX1 in ESCC tissues (Figure 1C,D). Subsequently, we evaluated the clinical significance of YBX1. The clinicopathological characteristics of patients with ESCC are shown in Table S1 (Supporting Information). We found that high levels of YBX1 were significantly associated with poor patient survival (Figure 1E). Together, these results provided evidence that elevated expression of YBX1 predicts poor prognosis in patients with ESCC.

### 2.2 YBX1 Facilitated the Proliferation, Invasion, and Pluripotency Maintenance of ESCC Cells In Vitro

**Figure 2:**
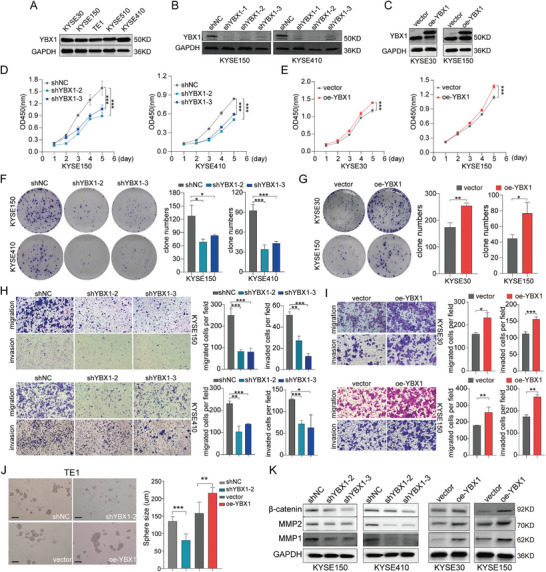
YBX1 promotes ESCC cells proliferation, migration, and invasion in vitro. A) YBX1 expression status in five ESCC cell lines was assessed by western blot. B,C) The efficiency of YBX1 knockdown and overexpression in ESCC cells was determined by a western blot assay. D–G) The proliferation ability of cells with YBX1 deficient and overexpression was examined by CCK‐8 and colony formation assays. H,I) Transwell assays of ESCC cells under YBX1 knockdown and overexpression. J) Sphere formation ability was determined in TE1 cells with YBX1 knockdown or overexpression, respectively. K) Protein expression status of *β*‐catenin, MMP1, MMP2 in YBX1 knockdown or overexpression ESCC cells. ^*^ *p* < 0.05, ^**^ *p* < 0.01, and ^***^ *p* < 0.001.

To determine the tumorigenic role of YBX1 in ESCC cells, we utilized YBX1‐specific shRNAs and an YBX1‐overexpressing plasmid to efficiently knockdown and overexpress YBX1, respectively (**Figure** **2**A–C). Interestingly, we detected that downregulation of YBX1 substantially decreased the proliferation of ESCC cells (Figure 2D,F), whereas ectopic expression of YBX1 showed the opposite effect (Figure 2E,G). To determine the biological function of YBX1 in ESCC metastasis, we further conducted cell migration and invasion assays. As shown in Figure 2H, both the migration and invasion abilities were significantly decreased in sh‐YBX1‐transfected ESCC cells compared with those transfected with sh‐NC. Conversely, we observed that ectopic expression of YBX1 markedly increased the migration and invasion of ESCC cells (Figure 2I).

Emerging evidence has shown that YBX1 is involved in maintaining the tumor stem cell phenotype.^[^ ^10^, ^14^ ^]^ Therefore, we evaluated the sphere‐forming ability of cells after knockdown or ectopic expression of YBX1. As shown in Figure 2J, YBX1 knockdown inhibited, whereas YBX1 overexpression promoted the sphere formation ability of TE1 cells. In addition, the expression level of proteins associated with epithelial to mesenchymal transition (EMT) and stemness, including MMP1, MMP2, and *β*‐catenin, was reduced by YBX1 knockdown, whereas it was significantly increased in ESCC cells overexpressing YBX1 (Figure 2K). These findings indicated that YBX1 may amplify the malignant phenotype of ESCC cells.

### 2.3 YBX1 is Required for In Vivo ESCC Growth and Metastasis

**Figure 3:**
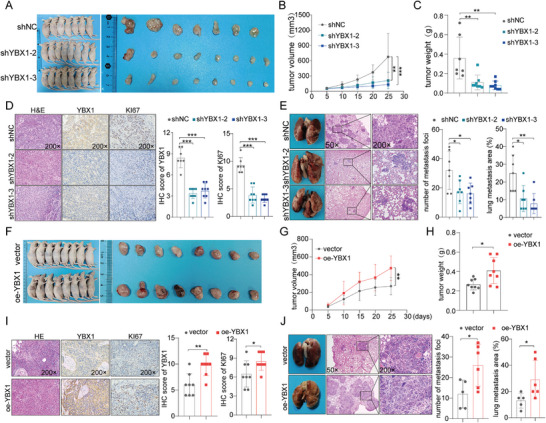
YBX1 facilitates xenograft tumor growth and lung metastasis of ESCC in vivo. A) Morphological pictures of the decreased subcutaneous xenograft tumor formation in mice injected with YBX1‐depleted KYSE150 cells. B,C) Tumor volume growth curves and final tumor weight were measured and quantified. D) Representative H&E and IHC images (left) of the tumor described. IHC staining data of YBX1 and KI67 of the mice in each group was quantified (right). E) Decreased tumor metastasis in mouse lungs with YBX1‐depleted KYSE150 cells, as determined by tail‐vein injection metastasis assays. Images of the mouse lungs with metastatic nodules and corresponding H&E staining of the metastatic tumors were presented and quantified for analysis. F) Morphological images of orthotopic‐xenograft mouse models implanted with YBX1‐overexpression in KYSE30 cells. G,H) Tumor growth and tumor weight were measured and quantified. I) H&E and IHC staining of the tumor (left). IHC staining data of YBX1 and KI67 of the mice in each group were quantified (right). J) Representative images of the overall observation of lungs with metastatic nodules and their corresponding H&E images (left). Lung metastasis nodules were further quantified (right). ^*^ *p* < 0.05, ^**^ *p* < 0.01, and ^***^ *p* < 0.001.

To determine the oncogenic function of YBX1 in vivo, xenograft mouse model was generated by subcutaneously injecting ESCC cells infected with sh‐YBX1 and sh‐NC. Consistent with the in vitro results, the tumor growth rate and average tumor weight in YBX1 knockdown groups were noticeably lower compared with those in the control group (**Figure** **3**A–C; Figure S1A–C, Supporting Information). Furthermore, IHC analysis confirmed that tumors generated from the YBX1 knockdown group showed weaker Ki‐67 staining than those from the control group (Figure 3D). An experimental model of pulmonary metastasis was used to assess the effect of YBX1 on ESCC metastasis. We observed that silencing YBX1 resulted in a markedly decreased number of lung metastatic nodules in nude mice compared with that in the control group, as revealed by the in vivo tail vein metastasis assay (Figure 3E). Conversely, YBX1 overexpression remarkably increased the rate of tumor growth and occurrence of lung metastasis in nude mice (Figure 3F–J). In summary, these findings further underscored the potential of YBX1 in promoting ESCC tumorigenesis and metastasis.

### 2.4 YBX1 Stabilizes *SMOX* mRNA in m5C‐Dependent Manner

**Figure 4:**
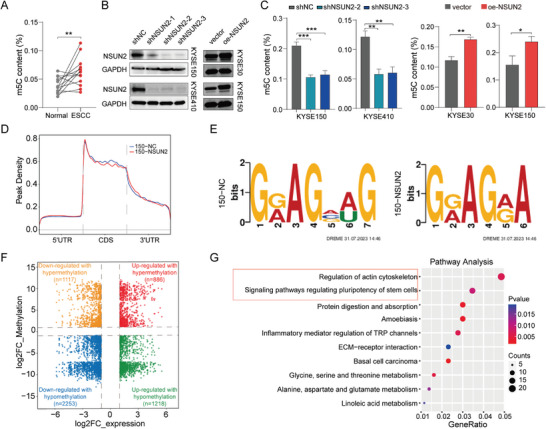
Characterization of mRNA 5‐methylcytosine (m5C) in ESCC. A) The mRNAs were extracted by two successive rounds of poly (A) purification using magnetic oligo d(T) beads from total RNAs. The levels of m5C in purified mRNA between ESCC tissues and adjacent normal esophageal tissues were measured by ELISA (n=13). B) The silencing and overexpression efficiency of NSUN2 was detected by western blot. C) m5C level in purified mRNA of ESCC cell lines with NSUN2 knockdown or overexpression was determined by ELISA. D) The m5C distributions within different regions in control and NSUN2 knockdown cells. E) The top consensus m5C motif in in control and NSUN2 knockdown cells. F) Distribution of mRNAs with a significant change in both the m5C methylation and gene expression levels in KYSE150 cells with NSUN2 knockdown. G) Pathway analysis of genes in cells with NSUN2 knockdown.

The levels of RNA m5C methylation in ESCC tissues and normal esophageal tissues were detected using ELISA. Accordingly, we found that the levels of mRNA m5C methylation were substantially upregulated in ESCC tissues (**Figures** **4**A; S2A, Supporting Information). We then performed differential analysis to screen for the hub m5C “writers” in ESCC using public datasets, and further validated them using our clinical ESCC specimens. Among them, NSUN2 drew our special attention because we detected its remarkably high expression in ESCC tissues and previous studies revealed that NSUN2 and YBX1 frequently cooperatively contributed to tumorigenesis (Figure S2B–E, Supporting Information). In addition, we detected that the level of m5C methylation was significantly reduced in *NSUN2* knockdown ESCC cells, whereas it was increased in *NSUN2*‐overexpressing ESCC cells (Figure 4B,C).

We next performed an m5C meRIP‐seq assay to investigate the effect of silencing *NSUN2* on the m5C profiles of ESCC cells. As reported previously,^[^ ^15^ ^]^ we found that the m5C methylation pattern was mainly distributed in the coding sequences (CDS) of mRNA transcripts in ESCC cells (Figure 4D). The preferred m5C consensus motif is shown in Figure 4E. The association of m5C modifications with gene expression were investigated and differentially expressed mRNAs (2253 down and 1218 upregulated) with hypomethylated m5C were identified (Figure 4F). Pathway analysis also revealed that genes in NSUN2‐deficient cells were significantly enriched in the regulation of actin cytoskeleton and signaling pathways regulating the pluripotency of stem cells (Figure 4G). Collectively, these findings suggested that NSUN2, an m5C methylase, plays a critical role in YBX1‐mediated ESCC progression.

**Figure 5:**
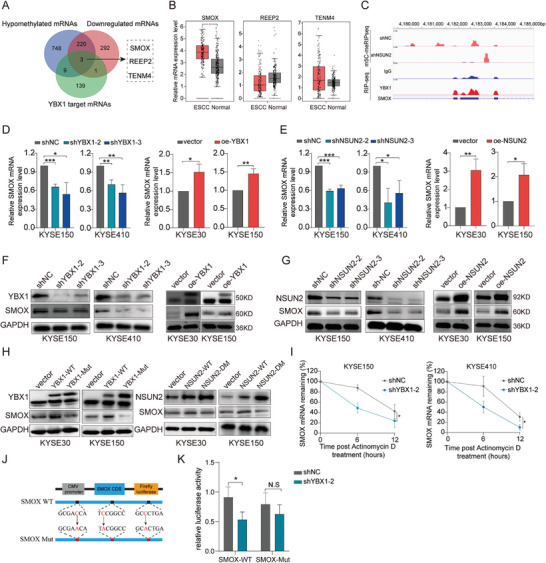
YBX1 enhances SMOX mRNA stability in an m5C‐dependent manner. A) Overlaying YBX1‐binding targets (Fold enrichment >10) with transcripts displaying reduced m5C sites (Foldchange >200) and expression level (logFC <−2) following NSUN2 knockdown in KYSE150 cells. B) mRNA expression level of *SMOX*, *REEP2*, and *TENM4* were evaluated in ESCA‐TCGA dataset. C) Integrative‐genomics‐viewer tracks displaying the m5C levels of *SMOX* in control and NSUN2‐depleted KYSE150 cells as well as the YBX1‐binding clusters in the RIP‐Seq data. D) *SMOX* mRNA expression levels in ESCC cells with YBX1 knockdown (left) or overexpression (right). E) SMOX mRNA expression levels in ESCC cells with NSUN2 knockdown (left) or overexpression (right). F) SMOX protein expression levels in YBX1 knockdown (left) or overexpression (right) ESCC cells. G) SMOX protein expression levels in NSUN2 knockdown (left) or overexpression (right) ESCC cells. H) Western blot analysis of SMOX expression level in ESCC cells transfected with pcDNA‐YBX1‐WT or pcDNA‐YBX1‐Mut (W65A) plasmid (left), and pcDNA‐NSUN2‐WT or pcDNA‐NSUN2‐Double Mut (C271A&C321A) plasmid (right). I) RNA stability assays in ESCC cells with YBX1‐depleted compared with the control cells by qRT‐PCR after administrated with actinomycin D at the indicated time points. J) SMOX CDS region containing wild or mutant (C‐to‐ A mutation) m5C sites was cloned into luciferase reporter vector. K) Relative luciferase activity of the wild‐type and mutant form of SMOX CDS reporter vectors in ESCC cells transfected with shNC or shYBX1, respectively. ^*^ *p* < 0.05, ^**^ *p* < 0.01, and ^***^ *p* < 0.001.

Subsequently, RNA immunoprecipitation sequencing (RIP‐seq) was performed to identify the downstream transcripts of YBX1 with m5C sites in ESCC. Combined with whole transcriptome m5C MeRIP‐seq and RNA‐seq analyses, we identified 223 mRNAs that exhibited significant downregulation of both mRNA expression and m5C methylation levels following *NSUN2* knockdown, three of which were directly bound by YBX1 (**Figure** **5**A). Intriguingly, we noticed that spermine oxidase (SMOX) was overexpressed in ESCC tissues (Figure 5B; Figure S3, Supporting Information). Integrative Genomics Viewer (IGV) analysis showed that the m5C level of SMOX was significantly decreased in KYSE150 cells after *NSUN2* knockdown, and that SMOX contained m5C sites in its CDS, which coincided well with the binding site of YBX1 (Figure 5C). In addition, we found that silencing YBX1 or *NSUN2* markedly downregulated, whereas ectopic expression of YBX1 or *NSUN2* significantly increased the levels of expression of SMOX, as identified by RT‐qPCR and western blot analyses (Figure 5D–G). Notably, WT YBX1/NSUN2, but not their mutants, upregulated the expression of the SMOX protein in ESCC cells (Figure 5H). These results strongly indicated that sustained expression of SMOX depends on the m5C catalytic activity of NSUN2 and m5C binding ability of YBX1. Considering the established critical function of YBX1 in mRNA stabilization, we validated the effect of YBX1 on the stability of *SMOX* mRNA. As expected, we found that following actinomycin D treatment, SMOX transcripts exhibited shorter half‐lives in YBX1 knockdown cells, whereas overexpression of YBX1 yielded the opposite results (Figure 5I), further underscoring that YBX1 could enhance the expression of *SMOX* by maintaining its mRNA stability. Based on the bioinformatics analysis that NSUN2‐regulated m5C modification regions are mainly in the CDS of *SMOX* mRNA, we constructed wild‐type (SMOX‐WT) and m5C sites mutant *SMOX* CDS (SMOX‐Mut) luciferase reporter plasmids (Figure 5J). We noticed that YBX1 knockdown led to significantly decreased luciferase activity in the SMOX‐WT group, but not in the SMOX‐Mut group (Figure 5K). These results indicated that YBX1 enhances the expression level of SMOX in an m5C‐dependent manner.

We further investigated whether the regulatory mechanism between YBX1 and SMOX depends on NSUN2‐mediated m5C modification. Results showed that both the malignancy‐promoting and positive regulatory effects on the expression of SMOX were substantially weakened in YBX1‐overexpressing cells with NSUN2 deficiency (Figure S4A‐D). Additionally, the m5C modification level was significantly correlated with NSUN2and YBX1 expression (Figure S4E, Supporting Information). Collectively, these data revealed that YBX1 can bind to and stabilize *SMOX* mRNA in a NSUN2‐mediated and m5C‐dependent manner.

### 2.5 NSUN2 Promotes ESCC Tumorigenesis and Progression

We evaluated the biological roles of NSUN2 in NSUN2‐deficient KYSE150 and KYSE410 cells. Functionally, we observed a striking decrease in the viability and clone numbers of NSUN2‐deficient ESCC cells compared with those in control cells (Figure S5A,C, Supporting Information). In contrast, ectopic expression of NSUN2 enhanced the proliferation of ESCC cells (Figure S5B,D, Supporting Information). In addition, in vitro assays revealed that silencing *NSUN2* significantly suppressed cell migration and invasion, whereas overexpressing *NSUN2* led to the opposite results (Figure S5E,F, Supporting Information). Collectively, these data indicated that NSUN2 promoted the proliferation, migration, and invasion of ESCC cells in vitro.

We then explored the effects of *NSUN2* knockdown on tumor growth and metastasis in vivo. We detected a slower growth rate, smaller size, and lower final weight of tumors in the NSUN2‐deficient group relative to those in the control group (Figure S5G–I, Supporting Information). Successful *NSUN2* knockdown was confirmed by IHC (Figure S5J, Supporting Information). Moreover, Ki‐67 staining showed that cell proliferation was impaired in tumors from the NSUN2‐deficient group (Figure S5J, Supporting Information). Consistently, H&E staining showed that *NSUN2* knockdown greatly decreased the lung metastatic colonization ability of ESCC cells (Figure S5K, Supporting Information). Taken together, our results showed that NSUN2 promotes ESCC tumorigenicity and metastasis in vivo.

### 2.6 YBX1/m5C‐SMOX Axis Promoted ESCC Progression Through Activating mTORC1 Signaling

**Figure 6:**
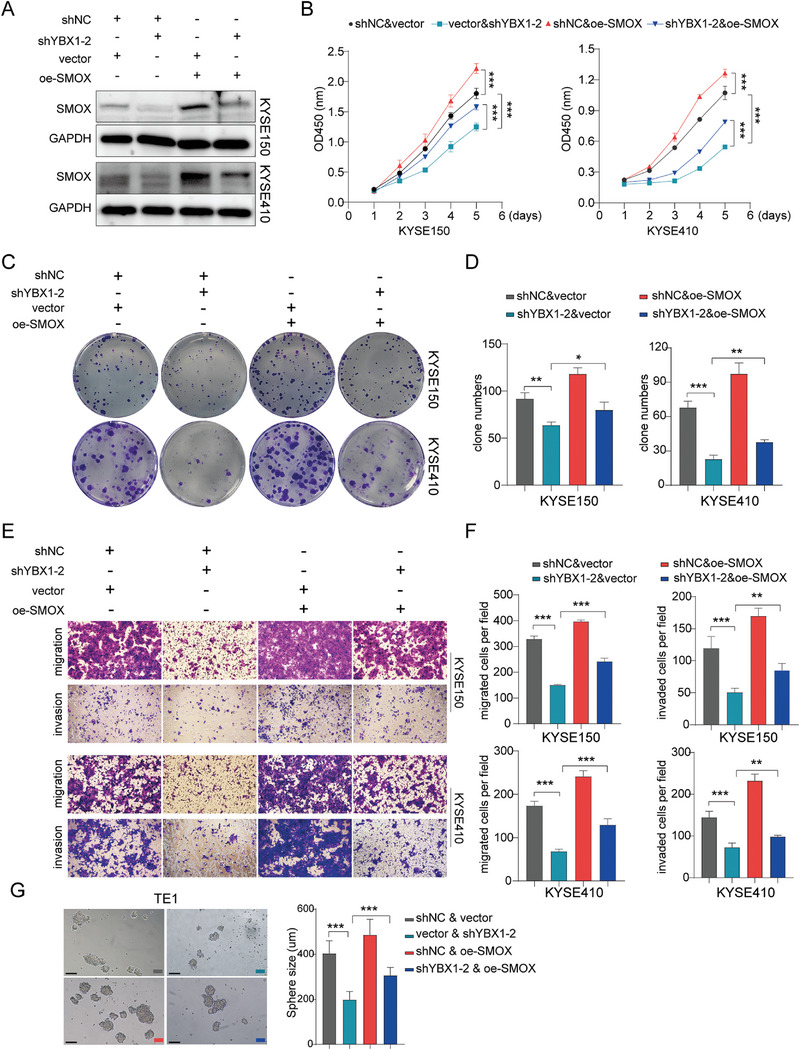
YBX1 assists ESCC cell proliferation, migration, and invasion partially through upregulating SMOX. A) The SMOX level was assessed through a western blot assay in ESCC cells co‐transfected with blank vector & sh‐NC, vector & shYBX1‐2, shNC & oe‐SMOX, or shYBX1‐2 & oe‐SMOX. B‐D) CCK8 and colony formation assay determined cell proliferation ability of YBX1 knockdown or control ESCC cells after co‐transfected with SMOX. E,F) Cell migration and invasion ability were evaluated by transwell assay and were further quantified. G) Sphere formation ability of scramble and shYBX1‐2 & oe‐SMOX co‐transfected cells. ^*^ *p* < 0.05, ^**^ *p* < 0.01, and ^***^ *p* < 0.001.

Considering our findings that YBX1 mediates the mRNA stabilization of SMOX in an m5C‐dependent manner, we postulated that the YBX1/SMOX axis plays a role in ESCC oncogenesis. To test this, we conducted rescue assays and found that reconstitution of SMOX‐WT but not SMOX‐MUT could partially recover the proliferation and metastasis ability of YBX1‐deficient cells (**Figure** **6**A–G; Figures S6A–F and S7A,B, Supporting Information). Overall, these data supported the notion that YBX1 promotes the metastasis and stemness maintenance of ESCC cells at least in part, by targeting SMOX.

**Figure 7:**
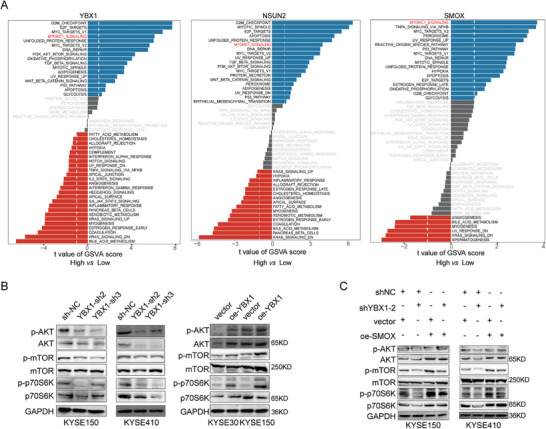
YBX1/m5C‐SMOX axis promotes ESCC progression through activating mTORC1 signaling. A) GSVA of ESCC samples with YBX1, NSUN2 and SMOX high expression *vs* low expression. B) Western blot assay to detect the protein level of p‐AKT, AKT, p‐mTOR, mTOR, p‐p70S6K and p70S6K in ESCC cell lines with YBX1 knockdown or overexpression, respectively. C) Western blot assay to determine p‐AKT, AKT, p‐mTOR, mTOR, p‐p70S6K, and p70S6K expression in scramble and shYBX1‐2 & oe‐SMOX co‐transfected cells.

Gene set variation analysis (GSVA) revealed that samples with high expression of YBX1/NSUN2/SMOX in the TCGA‐ESCC dataset were mainly enriched in the MTORC1_SIGNALING pathway (**Figure** **7**A). Further validation showed that YBX1 knockdown reduced the expression of p‐AKT, total AKT, p‐mTOR, and p‐p70S6K, whereas YBX1 overexpression led to the opposite results (Figure 7B). Moreover, the suppressive effects of p‐mTOR and pp‐70S6K were reversed by reconstitution with SMOX (Figure 7C). High level of p‐mTOR induced by overexpressing SMOX could be inhibited by rapamycin (Figure S8, Supporting Information). These results indicated that the YBX1‐mediated upregulation of SMOX may promote ESCC progression by activating the mTORC1 signaling pathway.

### 2.7 SMOX Expression was Positively Correlated with YBX1 and NSUN2 in ESCC

**Figure 8:**
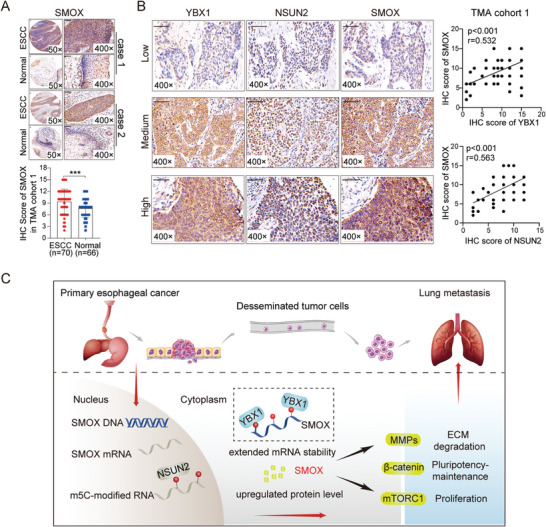
SMOX expression was positively correlated with YBX1 and NSUN2 in ESCC. A) Representative IHC staining images of SMOX in ESCC tissues and adjacent normal tissues (up). IHC staining data of SMOX was quantified (down). B) Representative IHC images (×400 magnification) of YBX1, NSUN2, and SMOX in ESCC tissues with low or high expression (left). Scale bars, 50 µm. Spearman's correlation analysis was performed (right). C) Proposed model illustrating the functional role of YBX1/m5C‐SMOX axis in facilitating the progression of ESCC.

Of note, TMA analysis revealed that the expression level of SMOX was increased in ESCC tissues (**Figure** **8**A). In addition, we observed a positive correlation between the levels of SMOX and expression of YBX1 (r = 0.532, p < 0.001) and NSUN2 (r = 0.563, p < 0.001) (Figure 8B). These data provided additional evidence that SMOX is a critical target of NSUN2/YBX1‐mediated m5C formation and recognition.

## 3 Discussion

Although abnormal RNA modifications have been associated with disease pathogenesis, including cancer tumorigenesis, the underlying relationship between ESCC and m5C remains poorly characterized. In this study, we verified that YBX1, an m5C reader protein that stabilizes the mRNA of target proteins, was upregulated in ESCC, promoting the proliferation and metastasis of ESCC cells. Furthermore, we confirmed that a YBX1‐mediated and m5C‐dependent mechanism was critical for the constitutive activation of SMOX in ESCC. Overall, we described the functional landscape of the YBX1/m5C‐SMOX axis in driving ESCC progression (Figure 8C). This study suggested potential prognostic biomarkers and highlighted the possibility of epitranscriptomic‐targeted therapies for ESCC.

Previous studies indicated the regulatory effect of m5C methylation on mRNAs, which is usually interpreted by reader proteins.^[^ ^16^ ^]^ As a multifaceted molecule, YBX1 functions as a transcription factor when located in the nucleus,^[^ ^17^ ^]^ whereas it typically serves as an RNA‐binding protein in the cytoplasm.^[^ ^10^ ^]^ Numerous studies have reported that YBX1 promotes tumor growth and metastasis in various cancer types including HCC,^[^ ^8^, ^18^ ^]^ colorectal cancer,^[^ ^9^ ^]^ and clear cell renal cell carcinoma.^[^ ^11^ ^]^ Notably, Chen et al. demonstrated a novel role of YBX1 in stabilizing the heparin‐binding growth factor (HDGF) mRNA by targeting the m5C‐modified site in bladder cancer.^[^ ^7a^ ^]^ In consistency with this, we found that the expression of YBX1 was frequently upregulated in human ESCC tissues and was mainly localized in the cytoplasm. Moreover, high levels of YBX1 enhanced the proliferation, migration, and invasion of ESCC cells in vitro. Consistently, the orthotopic tumor xenograft results further confirmed that YBX1 facilitated tumor proliferation and distant metastasis in vivo. These data indicated that YBX1 may play an oncogenic role in ESCC progression, at least in part, by relying on its m5C reader function.

We identified NSUN2 as a hub m5C writer protein that plays a crucial role in ESCC tumorigenesis. Emerging evidence has suggested that NSUN2 is the major methyltransferase that initiates m5C modification in mammalian mRNAs. In recent years, NSUN2 has attracted considerable attention because of its key role in driving the development of multiple tumors in an m5C‐dependent manner.^[^ ^19^ ^]^ *Sun* et al. demonstrated that NSUN2 promoted HCC development by mediating m5C modifications.^[^ ^20^ ^]^ Furthermore, NSUN2 enhanced the proliferation of gastric cancer cells by destabilizing the m5C‐modified *p57Kip2* mRNA.^[^ ^21^ ^]^ A recent study revealed that NSUN2‐mediated m5C modification of *GRB2* mRNA promoted ESCC progression.^[^ ^22^ ^]^ In accordance with previous studies, our study showed that NSUN2 was upregulated in ESCC tissues. In addition, high expression of NSUN2 led to increased levels of m5C‐modified mRNAs in ESCC cells, accelerating the proliferation and metastasis of ESCC cells. Taken together, these findings supported that NSUN2 is an essential methyltransferase responsible for YBX1‐mediated ESCC tumorigenesis.

We performed integrated analysis based on m5C MeRIP‐seq and RIP‐seq assays to gain a deeper insight into the oncogenic function of the NSUN2/YBX1/m5C axis in ESCC. We confirmed that YBX1 bound to and further stabilized *SMOX* transcripts, which underwent NSUN2‐catalyzed m5C modifications in their CDS region. SMOX is a polyamine catabolic enzyme that plays a vital role in carcinogenic signaling.^[^ ^23^ ^]^ Intriguingly, rescuing the expression of SMOX partially recovered the proliferation, migration, and invasion abilities of ESCC cells attenuated by YBX1 knockdown. Most importantly, both the YBX1‐induced malignancy‐promoting and positive regulatory effect on the expression of SMOX were substantially weakened by silencing *NSUN2*. Thus, our data revealed a potential mechanism by which the activation of SMOX is dependent on its m5C modification and recognition during the progression of ESCC.

SMOX plays an essential role in regulating tumorigenesis. For instance, *Sierra* et.al reported that SMOX participated in Helicobacter pylori‐induced *β*‐catenin activation in gastric cancer oncogenesis.^[^ ^24^ ^]^ The byproduct of the SMOX reaction produces the proinflammatory chemokine CXC motif ligand 1 (CXCL1), promoting cell migration.^[^ ^25^ ^]^ Consistently, we discovered that the YBX1/m5C‐SMOX axis enhanced the ESCC malignant phenotype and was involved in the regulation of proteins associated with EMT and cell stemness maintenance, including MMP1, MMP2, and *β*‐catenin. Notably, further experiments validated that YBX1 activated the mTOCR1 signaling pathway by targeting SMOX. Several studies have shown that the activation of the mTOCR1 signaling pathway is involved in ESCC tumor growth,^[^ ^26^ ^]^ autophagy,^[^ ^27^ ^]^ and therapeutic efficacy.^[^ ^28^ ^]^ These findings further corroborated the m5C‐mediated epigenetic regulatory mechanism by which the YBX1/m5C‐SMOX‐mTOCR1 axis is involved in the initiation and progression of ESCC.

In summary, the current study elucidated that YBX1 can promote ESCC progression by stabilizing *SMOX* mRNA and consequently activating the mTOCR1 signaling pathway in an NSUN2‐mediated m5C modification‐dependent manner. Our findings highlight the crucial role of YBX1 in tumorigenesis and metastasis of ESCC and provide a preclinical rationale for selectively targeting YBX1‐mediated m5C recognition as a promising therapeutic strategy for ESCC.

## 4 Experimental Section

### GEO and TCGA dataset

The gene expression data were obtained from the Genotype‐Tissue Expression (GTEx, https://gtexportal.org/home/), The Cancer Genome Atlas (TCGA, https://portal.gdc.cancer.gov), and Gene Expression Omnibus (GEO, https://www.ncbi.nlm.nih.gov/geo/), including GTEx, TCGA‐ESCA, GSE5364, GSE20347, GSE23400, GSE38129, GSE92396, GSE13898, and GSE89102 cohorts. Gene Expression Profiling Interactive Analysis (GEPIA, http://gepia.cancer‐pku.cn/) was utilized to evaluate the mRNA expression level of SMOX, REEP2, and TENM4. Detailed information of GEO datasets enrolled in this research was shown in Table S2 (Supporting Information).

### Clinical Specimens

Sixteen pairs fresh ESCC tissues and para‐cancer tissues were sourced between May 2020 and October 2020, at the First Affiliated Hospital of Zhengzhou University. ESCC tissue microarray (TMA) cohort 1 including 70 ESCC tissues and 66 normal tissues were sourced from the First Affiliated Hospital of Zhengzhou University. TMA cohort 2 including 108 ESCC tissues and 72 para‐cancer tissues were got from OUTDO Biotech Corp (Shanghai, China). This study was approved by the Ethical Review Committees of the First Affiliated Hospital of Zhengzhou University.

### Cell Lines and Cell Culture

Five human ESCC cell lines including TE1, KYSE30, KYSE410, KYSE150, and KYSE510, were obtained from the Shanghai Institute for Biological Science, Chinese Academy of Science (Shanghai, China). Cells were maintained in RPMI 1640 medium (VivaCell, Shanghai, China) supplemented with 10% fetal bovine serum (VivaCell, Shanghai, China) and cultured in an atmosphere of 5% CO2 at 37 °C.

### Lentivirus Infection and Plasmid Transfection

Lentivirus vectors were generated by Hanheng Technology Corp (Shanghai, China). YBX1 and NSUN2 knockdown lentiviruses were termed as shYBX1, shNSUN2 respectively, and the control lentivirus was termed as shNC. YBX1 and NSUN2 overexpression lentiviruses were termed as oe‐YBX1 and oe‐NSUN2, and a negative control was termed as vector. Lentivirus infection was performed as previously described.^[^ ^29^ ^]^ PCMV‐YBX1‐WT, PCMV‐YBX1‐Mut (W65A), pcDNA3.1‐NSUN2‐WT, pcDNA3.1‐NSUN2‐Mut (C271/321A), plasmids were kindly provided by Dr. Xiangyi Sun. (Department of Pharmacy, the First Affiliated Hospital of Zhengzhou University).

### Western Blot

RIPA lysis buffer (Solarbio, China) containing protease inhibitor (MCE, China) was utilized to isolate the total protein from tissue or cells. Then the boiled lysates were separated and transferred to PVDF membranes (Millipore, USA). After blocking, the membranes were incubated with specific primary and corresponding secondary antibodies, and finally visualized by Odyssey Infrared Imaging System (LI‐COR Bioscience, NE) or LumiGLO enhanced chemiluminescent (ECL). Antibodies used here were listed in Table S3 (Supporting Information).

### Immunohistochemistry (IHC)

The details of IHC were previously described.^[^ ^30^ ^]^ Cells containing brown granules were scored according to the proportion of positive cells: 0, none; 1, <20%; 2, 21–40%; 3, 41–60%; 4, 61–80%; and 5, 81–100%. The staining intensity was scored as follows: 0, none; 1, weak; 2, moderate; and 3, strong. The total staining score (range 0–15) was calculated by multiplying the two subscores. IHC staining index < or ≥10 was identified as cutoff value for protein low or high expression. Antibody details were shown in Table S3 (Supporting Information).

### RNA Extraction, cDNA Synthesis, and Quantitative PCR

Total RNAs were isolated and cDNA was generated by reverse transcription according to the protocol of RevertAid First Strand cDNA Synthesis Kit (Thermo Scientific). Then qRT‐PCR was performed using SYBR Green SuperMix (Roche) on LightCycler 96 (Roche, USA). Relative gene expression level was determined using the 2^−△△CT^ method. GAPDH was used to normalize mRNA expression. The detailed primer sequences are presented in Table S4 (Supporting Information).

### Cell Proliferation Assay

Cell proliferation was evaluated using CCK‐8 (Dojindo, Japan). Briefly, 4000 cells were seeded into 96 well plates. Cell viability was measured on a microplate reader. For clone formation assay, 600–800 cells were seeded and cultured for 10–14 days. After fixing and staining, the number of colonies was finally determined.

### Transwell Assay

For the transwell assay, 1 × 10^5^ infected cells were seeded into the upper chamber (coated or uncoated with Matrigel) in 200 µL serum‐free medium, and 600 µL medium with 10% FBS was added to the lower chamber. After incubation of 36 h, cells were fixed and stained. Three visual fields were randomly selected and photographed using a microscope (Olympus, Japan).

### Sphere Formation Assay

For the sphere formation assay, 2000 cells were cultured in 1 mL DMEM/F12 Medium (Gibco) supplemented with 2% B27 supplement (Gibco), 20 ng mL^−1^ human bFGF (PeproTech), 20 ng mL^−1^ Human EGF (PeproTech), 4 µg mL^−1^ Heprin (sigma) and 1% penicillin/streptomycin solution (Solarbio) in Nunclon Sphera 24‐Well Plate. Spheres were counted under the microscope after 7 days incubation. Images were taken at 10X magnification.

### Mouse Xenograft Model and Lung Metastasis Model

KYSE150 cells (5 × 10^6^) were subcutaneously injected into the right flanks of BALB/c nude mice (male, 5‐weeks‐old, 8 mice per group) to establish xenograft nude mice model. The volume of tumor was measured every 5 days. After 25 days, the mice were sacrificed, and the tumors were separated and fixed in 4% formalin for subsequent examination.

For mouse lung metastasis model, KYSE150 or KYSE30 cells (1 × 10^6^) were intravenously injected into nude BALB/c mice (male, 6‐weeks‐old). After six weeks, the mice were all euthanized, and their lungs were fixed in a 4% formalin solution. Tumor lesions within the lung tissues were confirmed through H&E staining. The lung metastatic foci were counted. Animal studies were approved by the Animal Research Ethics Committee of Zhengzhou University. All animal experiments were strictly implemented in compliance with the NIH Guide for the Care and Use of Laboratory Animals.

### RNA Methylation ELISA Assay

Total RNA was isolated and global m5C RNA methylation levels was detected using a MethylFlash 5‐mC RNA Methylation ELISA Easy Kit (EpiGentek, USA). Generally, After RNA binding and washing, the m5C detection complex solution was added to the wells and incubate at room temperature. After washing, fluorescence development solution was added and the signal was detected at 530_ex_ /590_em_ nm.

### M5C Dot Blot Analysis

The purified mRNA was mixed with RNA loading buffer and dropped onto the Hybond‐N+ membrane (Solarbio), followed by UV crosslinking. After blocking, the membranes were incubated with anti‐m5C antibody (ab214727, Abcam) and corresponding secondary antibodies and finally visualized by the ECL system. Equal RNA loading was verified by methylene blue (MB) staining.

### RIP‐Seq Assay

RIP assay was conducted with GenSeq RIP Kit. Briefly, cells lysates were incubated with specific antibodies against YBX1 (ab76149, Abcam). IgG was used as an internal control. After removing rRNA, coimmunoprecipitated RNA and input RNA were subjected to construct RNA sequencing library using NEBNext Ultra II Direct RNA Library Prep Kit. The library sequencing was performed on Illumina NovaSeq 6000 with 150 bp paired‐end reads. And the data to GEO (GSE249719) have uploaded.

### M5C MeRIP‐Seq Assay

Cloudseq Biotech Inc. (Shanghai, China) provided high‐throughput RNA m5C MeRIP‐sequencing. Briefly, after coimmunoprecipitating with specific m5C antibodies, the input RNAs and immunoprecipitated RNAs with m5C modified sites were used to construct RNA libraries with the TruSeq Stranded Total RNA Library Prep Kit (Illumina, America) according to the manufacturer's instructions. Library sequencing was performed on an Illumina NovaSeq instrument with 150 bp paired‐end reads. And the data to GEO (GSE249045) have uploaded.

### RNA Stability Assay

To assess the half‐life of SMOX mRNA, 5 µg ml^−1^ actinomycin‐D (Sigma, USA) was added into cell culture medium. At diverse time points, cells were collected and total RNA was isolated for qRT‐PCR analysis.

### Reporter Gene Assays

CDS‐SMOX‐wild type or CDS‐SMOX‐mutant (C855/1035/1135A) luciferase reporter plasmids were synthesized and cloned into GV712 vector by Genechem (Shanghai, China). Cells were seeded into 96‐well plates and subsequently transfected with 200 ng luciferase reporter plasmid and 20 ng pRL‐TK Renilla luciferase reporter. Luciferase activity in total cell lysates was measured using a Dual‐Glo Luciferase Assay System (Promega).

### Statistical Analysis

Statistical analysis was performed using R and GraphPad Prism 8 software. The differences between two independent groups were evaluated using Student's *t*‐test (unpaired, two‐tailed) or repeated measure variance analysis. A Kaplan–Meier overall survival analysis were performed with a log‐rank test. The Pearson's rank correlation test was utilized to perform the correlation analysis P<0.05 indicated statistically significant differences.

## Conflict of Interest

The authors declare no conflict of interest.

## Author Contributions

L.L., Y.C., T.Z., and G.C. contributed equally to this work. S.R. and X.W. designed the study. L.L., C.Y., Z.T., C.G., and Z.G. performed experiments and interpreted the data. W.W., W.Y., and Z.Y. collected surgically removed ESCC tissues. L.J. conducted the bioinformatic analysis. L.L. wrote the manuscript. S.R., R.Z., and W.Y. reviewed the manuscript and made significant revisions on the drafts. All authors read and approved the final manuscript.

## Supporting information

Supporting Information

## Data Availability

The data that support the findings of this study are available from the corresponding author upon reasonable request.
