# Supplementary material for: YBX1 Promotes Esophageal Squamous Cell Carcinoma Progression via m5C‐Dependent SMOX mRNA Stabilization
Source: Adv Sci (Weinh). 2024 Apr 2;11(20):2302379. [Article in Italian] doi: 10.1002/advs.202302379 (PMC11132058; doi:10.1002/advs.202302379)
Supplement: Supplementary file 1 — Supporting Information [file ADVS-11-2302379-s001.pdf]

ADVANCED SCIENCE
Open Access

Supporting Information

for *Adv. Sci.*, DOI 10.1002/advs.202302379

YBX1 Promotes Esophageal Squamous Cell Carcinoma Progression via m5C-Dependent SMOX mRNA Stabilization

Liwen Liu, Yu Chen, Tao Zhang, Guangying Cui, Weiwei Wang, Guizhen Zhang, Jianhao Li, Yize Zhang, Yun Wang, Yawen Zou, Zhigang Ren, Wenhua Xue* and Ranran Sun**

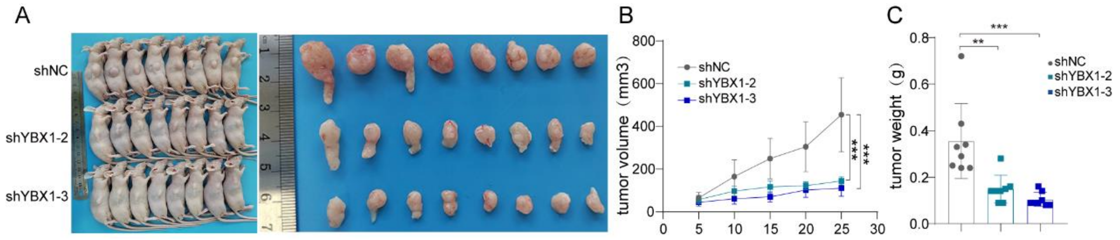

Figure S1. YBX1 facilitates xenograft tumor growth of ESCC in vivo. A) Morphological pictures of the decreased subcutaneous xenograft tumor formation in mice injected with YBX1-depleted KYSE410 cells. B-C) Tumor volume growth curves and final tumor weight were measured and quantified. **P < 0.01, and ***P < 0.001.

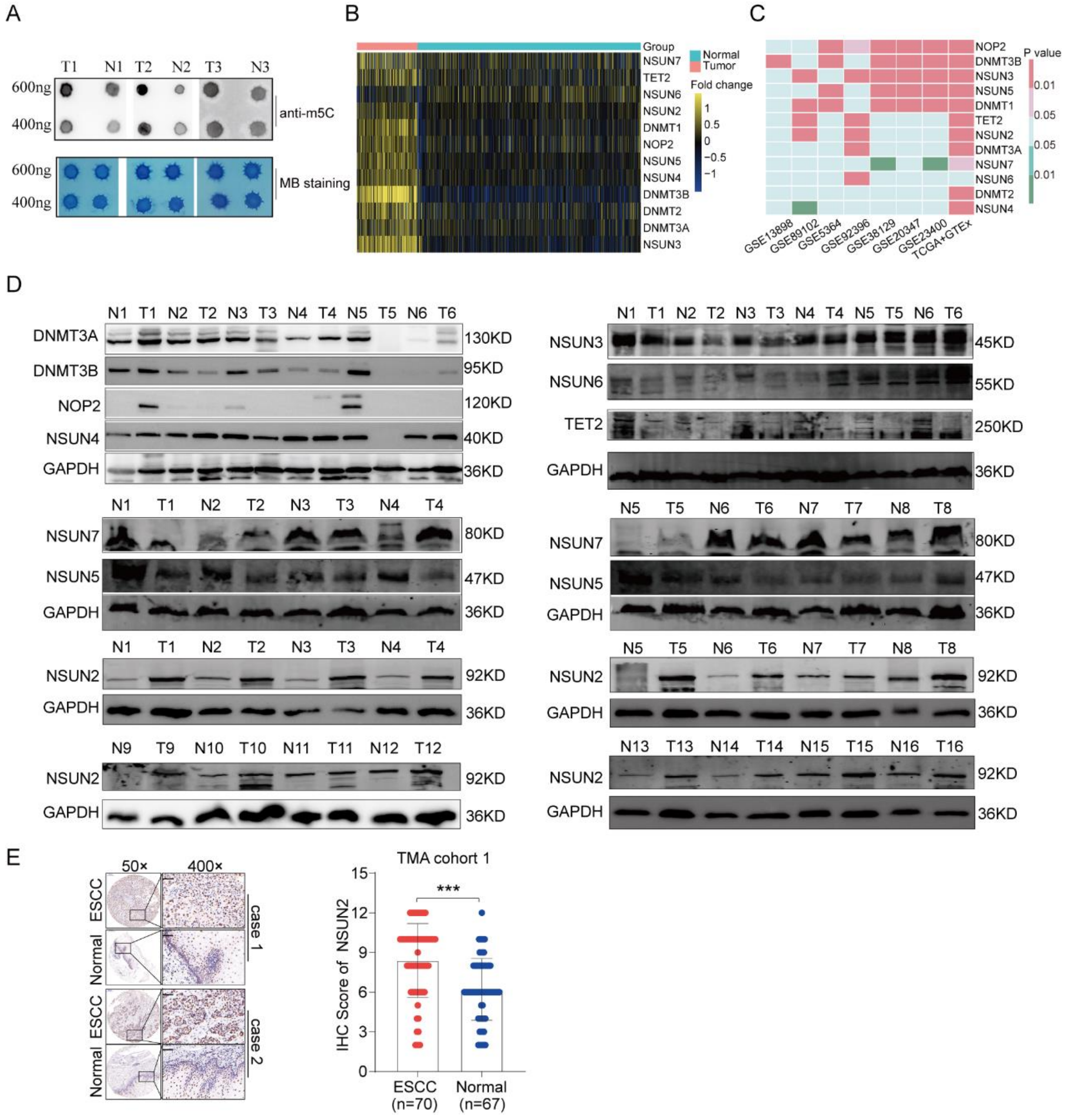

Figure S2. NSUN2 was remarkably upregulated in ESCC. A) The m5C level in ESCC tissues and adjacent nontumor tissues was detected by dot blot. B-C) Differentially expressed genes of the known m5C methyltransferases in TCGA, GTEx, and GEO database. D**)** Western blot assay to determine the expression level of m5C methyltransferases and demethylases in ESCC tissues. E) Representative IHC images of NSUN2 in ESCC tissues and adjacent normal tissue (left). IHC staining data of NSUN2 in ESCC tissues and normal tissue was quantified (right). ***P < 0.001.

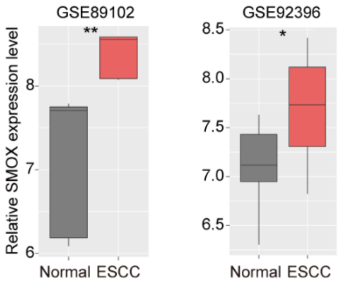

Figure S3. The mRNA expression level of SMOX was validated in GEO datasets.

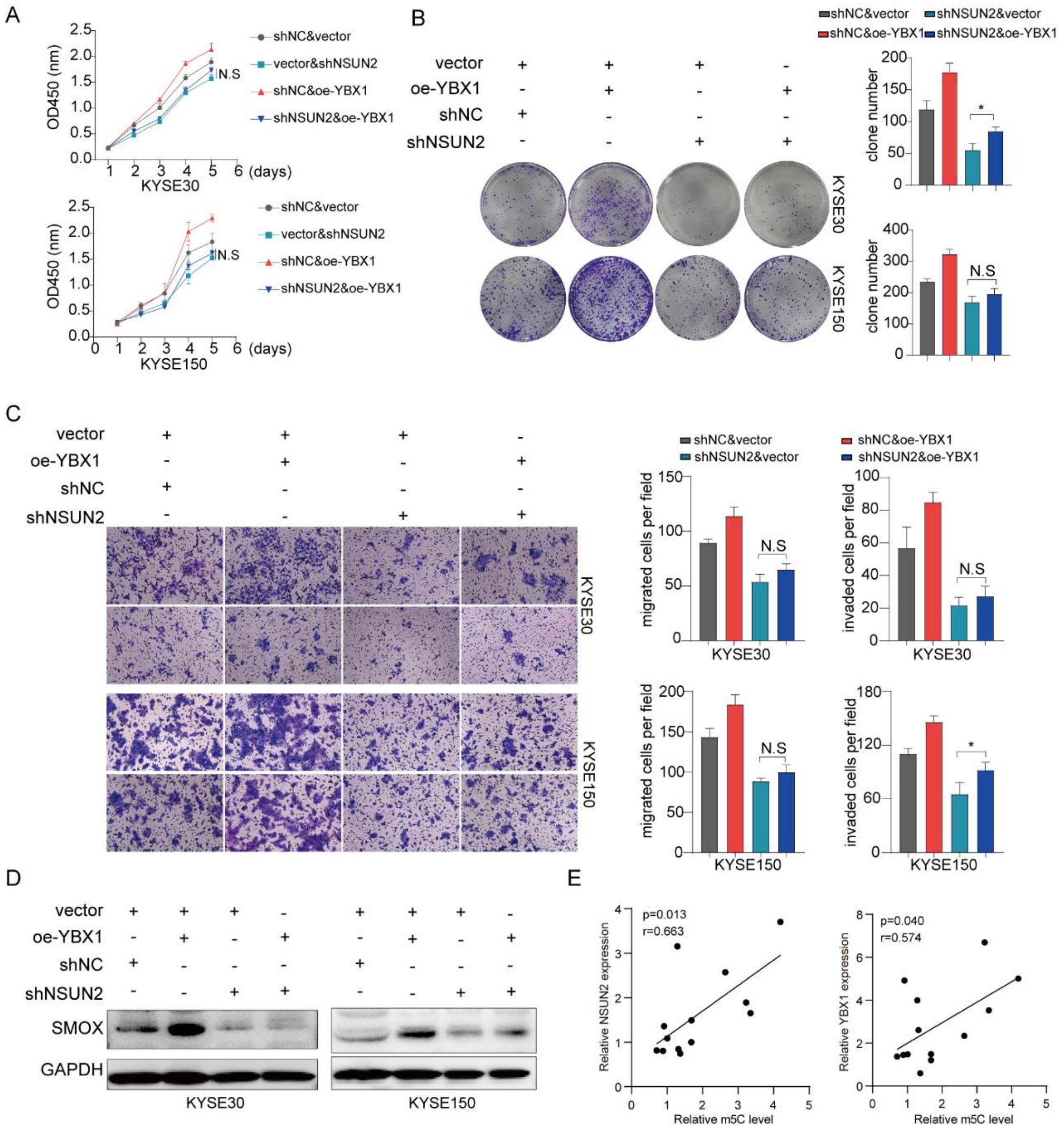

Figure S4. NSUN2 facilitates YBX1-mediated ESCC cell proliferation, migration, and invasion. A-B) The proliferation ability of ESCC cells co-transfected with blank vector & sh-NC, vector & shNSUN2, shNC & oe-YBX1, or shNSUN2 & oe-YBX1 was determined via CCK-8 and colony formation assays. C) Transwell assay of ESCC cells co-transfected with blank vector & sh-NC, vector & shNSUN2, shNC & oe-YBX1, or shNSUN2 & oe-YBX1. D) The expression level of SMOX was detected by western blot assay. E) Correlation analysis between the m5C RNA modification levels and the expression levels of YBX1/NSUN2. $*P<0.05$.

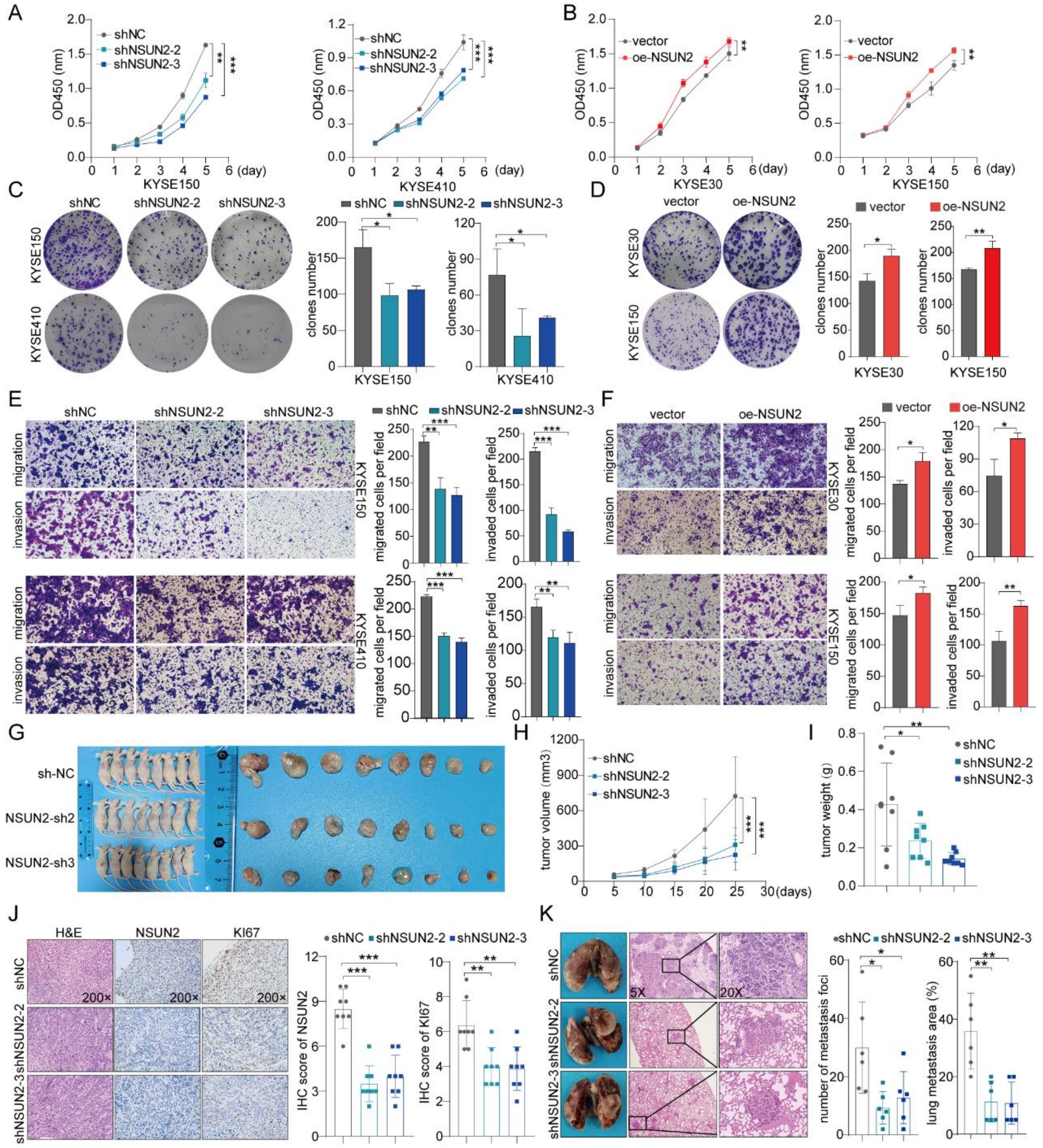

Figure S5. NSUN2 facilitates ESCC growth and metastasis both in vitro and in vivo. A-D) The proliferation ability of ESCC cells with NSUN2 silencing or overexpression was assessed by CCK-8 and colony formation assays. E-F) Representative images of transwell assay with ESCC cells under NSUN2 silencing and overexpression (left). The migrated and invaded cells are displayed in the right panel. G) Morphological images of reduced subcutaneous xenograft tumor formation

in mice injected with NSUN2-deficient KYSE150 cells. H-I) Tumor growth curves and tumor weight were measured and quantified. J) Representative H&E and IHC staining images of the tumor described (left). IHC staining data of NSUN2 and KI67 of the mice in each group quantified (right). K) Representative images illustrating the general observation of lungs with metastatic nodules, along with corresponding H&E images of the tumor edges in lungs, are presented on the left. The metastatic lung nodules were quantified on the right. Data was reported as mean ± standard deviation (SD). **P* < 0.05, ***P* < 0.01, and ****P* < 0.001.

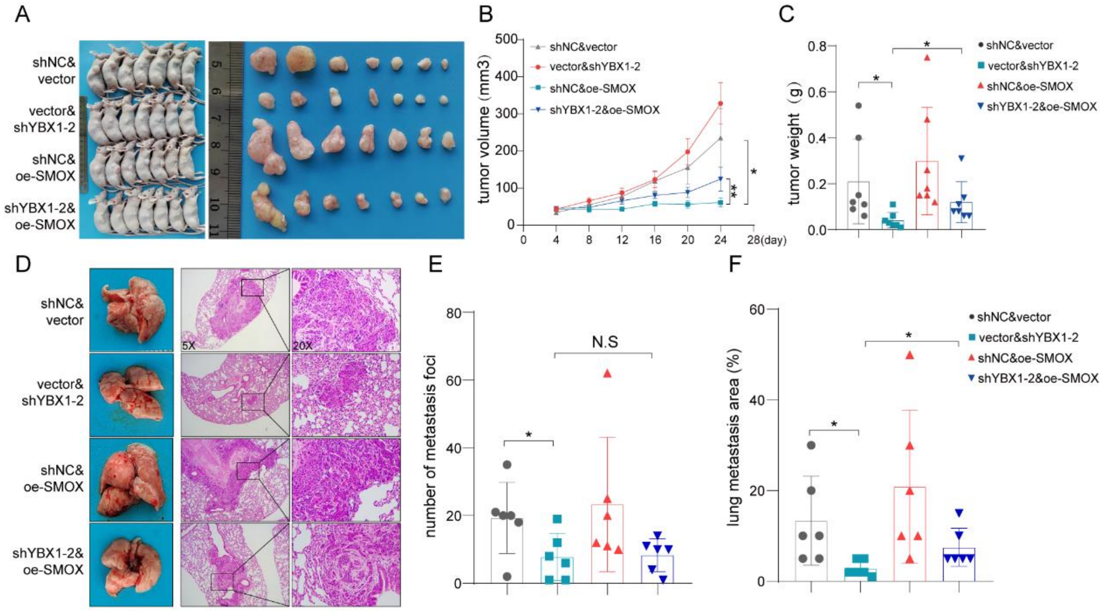

Figure S6. Reconstitution of SMOX partially recovered the proliferation and metastasis ability of ESCC cells curbed by YBX1 knockdown. A) Morphological pictures of the subcutaneous xenograft tumor formation in mice injected with ESCC cells co-transfected with blank vector & sh-NC, vector & shYBX1-2, shNC & oe-SMOX, or shYBX1-2 & oe-SMOX. B-C) Tumor volume growth curves and final

tumor weight were measured and quantified. D) Representative images of the overall observation of lungs with metastatic nodules and their corresponding H&E images. E-F) Lung metastasis nodules were further quantified. N.S not significant. *P < 0.05, and **P < 0.01.

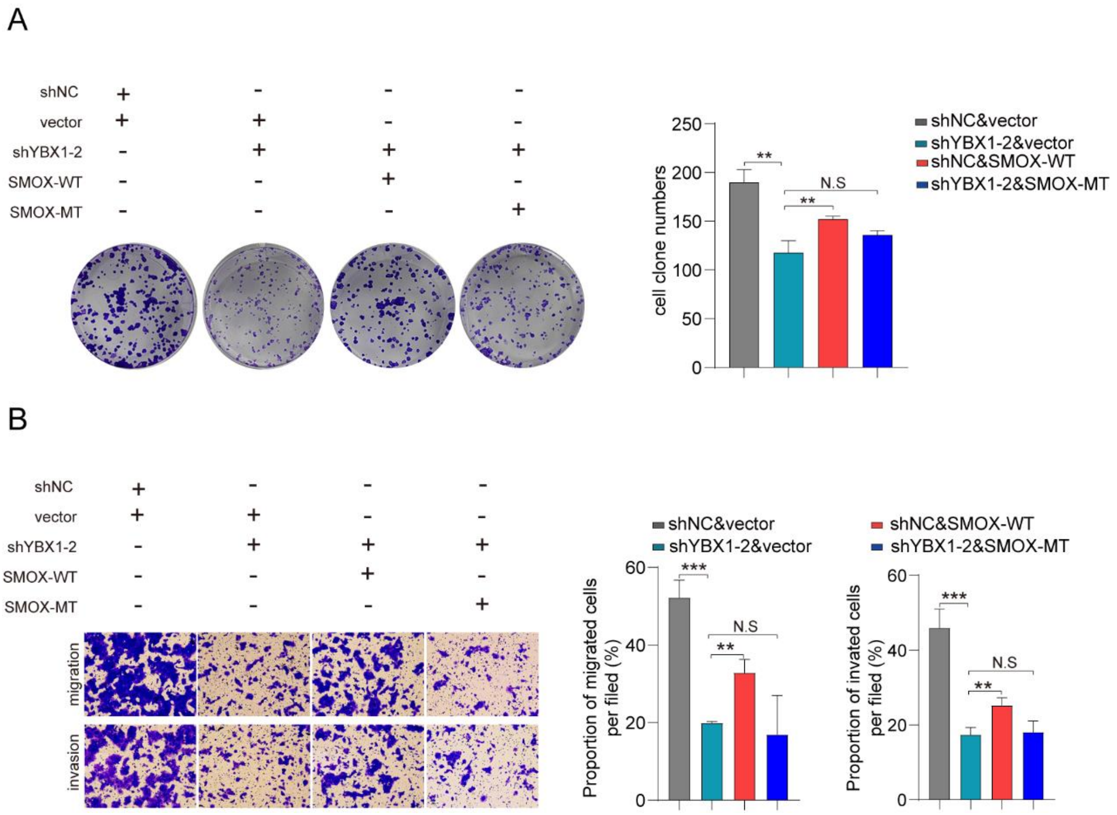

Figure S7. Reconstitution of SMOX-WT but not SMOX-MT could partially recover the proliferation (A) and metastasis (B) ability of ESCC cells curbed by YBX1 knockdown. **P < 0.01, and ***P < 0.001.

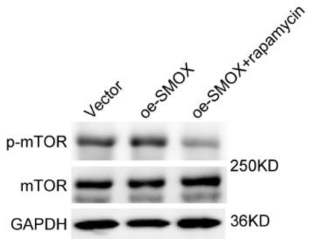

Figure S8. Overexpression of SMOX induced high level of p-mTOR could be inhibited by rapamycin.

Table S1 Clinico-pathological characteristics of ESCC patients

Clinico-pathological characters		Number. of cases (n)	YBX1 expression		X^2	*P Value*
			Low	High		
Age (years)	≤60	30	18	12	0.189	0.664
	>60	77	32	45		
Gender	Male	82	37	45	2.949	0.086
	Female	26	13	13		
Tumor size	T1-2	20	11	9	0.797	0.372
	T3-4	82	36	46		
Node	N0	35	17	18	0.507	0.476
	N1-2	42	17	25		
TNM stage	Stage I-II	49	24	25	0.212	0.645
	Stage III-IV	54	24	30		

Table S2 GEO microarray data enrolled in this study

Accession number	Platform	Number of samples		Country	Years
		Non-tumor	ESCC		
GSE5364	Affymetrix	13	16	Singapore	2018
GSE20347	Affymetrix	17	17	USA	2018
GSE23400	Affymetrix	104	104	USA	2019
GSE38129	Affymetrix	30	30	USA	2018
GSE92396	Affymetrix	9	12	USA	2018
GSE89102	Agilent	5	5	China	2016
GSE13898	Illumina	28	64	USA	2011
Total		206	248		

Table S3 Information on antibodies used in this study

Antibody	IHC	Western blot	Specificity	Company
YBX1	1:200	1:1000	Rabbit	Abcam
NSUN2	1:200	1:4000	Rabbit	Proteintech
SMOX	1:200	1:1000	Rabbit	Proteintech
GAPDH	/	1:5000	Mouse	Proteintech
NOP2	/	1:1000	Rabbit	Proteintech
NSUN3	/	1:1000	Rabbit	Abclonal
NSUN4	/	1:1000	Rabbit	Abclonal
NSUN5	/	1:1000	Rabbit	Abclonal
NSUN6	/	1:1000	Rabbit	Proteintech
NSUN7	/	1:800	Rabbit	Proteintech
DNMT3A	/	1:5000	Rabbit	Proteintech
TET2	/	1:1000	Rabbit	Proteintech
β-catenin	/	1:5000	Rabbit	Proteintech
MMP1	/	1:1000	Rabbit	Proteintech
MMP2	/	1:800	Rabbit	Abcam
AKT	/	1:1000	Mouse	Proteintech
P-AKT	/	1:1000	Mouse	Proteintech
mTOR	/	1:1000	Mouse	Proteintech
p-mTOR	/	1:1000	Mouse	Proteintech

P70S6K	/	1:1000	Mouse	Proteintech
P-P70S6K	/	1:1000	Rabbit	Proteintech

Table S4 Primer sequences used in this study

Gene name		Primer sequences
SMOX	Forward	5’-GGCGACCACAATCACGACACTG-3’
	Reverse	5’- ACCACCGACCACTGCTCATCC-3’
GAPDH	Forward	5’- GAACGGGAAGCTCACTGG -3’
	Reverse	5’- GCCTGCTTCACCACCTTCT -3’
